# Acute Liver Failure and Acute-on-Chronic Liver Failure in COVID-19 Era

**DOI:** 10.3390/jcm11144249

**Published:** 2022-07-21

**Authors:** Tatsuo Kanda, Reina Sasaki-Tanaka, Tomotaka Ishii, Hayato Abe, Masahiro Ogawa, Hirayuki Enomoto

**Affiliations:** 1 Division of Gastroenterology and Hepatology, Department of Medicine, Nihon University School of Medicine, 30-1 Oyaguchi-kamicho, Itabashi-ku, Tokyo 173-8610, Japan; sasaki.reina@nihon-u.ac.jp (R.S.-T.); ishii.tomotaka@nihon-u.ac.jp (T.I.); ogawa.masahiro@nihon-u.ac.jp (M.O.); 2 Department of Digestive Surgery, Nihon University School of Medicine, 30-1 Oyaguchi-kamicho, Itabashi-ku, Tokyo 173-8610, Japan; abe.hayato@nihon-u.ac.jp; 3 Division of Hepatobiliary and Pancreatic Disease, Department of Internal Medicine, Hyogo Medical University, Mukogawa-cho 1-1, Nishinomiya 663-8501, Japan; enomoto@hyo-med.ac.jp

Acute liver failure (ALF) and acute-on-chronic liver failure (ACLF), respectively, occur in patients with normal liver and patients with chronic liver diseases, including cirrhosis [1]. In general, both syndromes possess poor prognosis. The etiology of liver failure, such as hepatitis A virus (HAV), hepatitis B virus (HBV), hepatitis D virus (HDV) or hepatitis E virus (HEV), drugs, autoimmune hepatitis (AIH) and others, varies in various countries [1,2,3,4]. Although liver failure is currently a common medical disease, its incidence is increasing with the use of alcohol and with the growing epidemic of obesity and diabetes, leading to increases in the incidence of ACLF [4,5,6]. In this editorial, we discuss the recent progress regarding research on ALF and ACLF in the coronavirus disease 2019 (COVID-19) era (Figure 1).

In the COVID-19 era, severe acute respiratory syndrome coronavirus 2 (SARS-CoV-2) infection is also an important acute insult in ACLF patients [7]. To some extent, hepatocytes and biliary epithelial cells express the angiotensin-converting enzyme 2 (ACE2) receptor, which is one of the receptor candidates for SARS-CoV-2 [8]. COVID-19 infections may contribute to both primary and secondary liver injuries in patients with or without pre-existing liver diseases, respectively, leading to ALF or exacerbation of underlying liver diseases and ACLF [8]. In younger women, female sex hormones are protective in this regard [8]. A Fibrosis-4 (FIB-4) score above the threshold of 3.25 suggests the presence of liver fibrosis and is associated with higher mortality in people hospitalized with COVID-19 infections [9]. These patients may be associated with previously undocumented liver diseases, fibrosis and/or quiescent metabolic associated fatty liver diseases (MAFLD), and undiagnosed non-alcoholic steatohepatitis (NASH) (Figure 1) [9,10].

In patients with COVID-19, drug-induced liver injury (DILI) has often been observed (Figure 1). In total, 10.9% patients with COVID-19 were found to have DILI [11]. The frequency of DILI in patients who recovered from COVID-19-induced hepatitis was 36.2% [11]. The most commonly associated drugs were hydroxychloroquine, azithromycin, tocilizumab and ceftriaxone [11]. Delgado et al. reported that remdesivir had the highest incidence of DILI per administration [11].

Although a recent study [12] reported that liver injury in patients infected with COVID-19 did not seem to be associated with a higher risk of mortality, these results may be associated the distribution of COVID-19 vaccination or the SARS-CoV-2 Omicron variant. Further studies will be needed. Patients with chronic liver diseases should be vaccinated against COVID-19, and special attention for COVID-19 should be paid to patients with liver diseases [9,13].

AIH was occasionally observed after COVID-19 vaccination (i.e., vaccine-induced AIH) (Figure 1) [14,15]. A recent study indicated fast uptake of the COVID-19 mRNA vaccine BNT162b2 into human liver cell line Huh7, leading to changes in the expression and distribution of long interspersed nuclear element-1 (LINE-1), which is an endogenous reverse transcriptase, and that BNT162b2 mRNA is reverse transcribed intracellularly into DNA in as fast as 6 h upon BNT162b2 exposure. Thus, the COVID-19 mRNA vaccine is able to enter the human liver cell line Huh7 in vitro [16]. The use of immunosuppressants has been correlated to an increase in autoimmune liver disease severity and to lower levels of anti-SARS-CoV-2 antibodies upon vaccination [15]. All of the cases with AIH and post-COVID-19 vaccination have been successfully treated with steroids [15]. The assessment of low-density granulocytes (LDGs) may turn out to be a useful marker in the diagnosis of AIH [17].

The outbreak of acute severe hepatitis of unknown origin in children has recently been reported [18]. Some cases have tested positive for human adenoviruses and/or SARS-CoV-2 infection. Pediatric ALF differs from adult ALF, according to the type, the diversity of causes and the late appearance of hepatic encephalopathy [19]. In pediatric ALF, 20% of those who never developed hepatic encephalopathy died or underwent liver transplantation. Currently, 10–15% of liver transplantation indications in children are in ALF patients [19]. Finding the best-predicting score in pediatric ALF and early referral of the children to a specialized center are the most important issues (Figure 1) [19].

In certain cases, bacterial infection is also related to the development of ACLF. Takaya et al. reported that endotoxin level was a predictive factor independently associated with ACLF development [20]. They also showed that rifaximin decreased the endotoxin level and the risk of ACLF development in Child–Pugh class B, Japanese cirrhotic patients [20]. Endotoxin concentration was determined in whole blood by luminol chemiluminescence using a commercially available semiquantitative endotoxin activity assay [20]. Endotoxin, a lipopolysaccharide, is derived from the outer membrane of Gram-negative bacteria, and lipopolysaccharide (LPS) was recognized by Toll-like receptors (TLRs) of the liver, resulting in the activation of innate immune responses and the development of liver failure to some extent [20,21]. Endotoxin levels as well as Child–Pugh scores reflect the functional liver capacity and are independently associated with the development of ACLF in cirrhotic patients.

A meta-analysis of published studies on patients following liver resection for hepatocellular carcinoma (HCC) demonstrated that albumin-bilirubin (ALBI) grades 2 and 3 showed increased rates of post-hepatectomy liver failure compared with patients with grade 1 ALBI 1 [22]. ALBI grade is a useful liver-function assessment method in the systemic treatment for HCC patients [23]. ALBI grade is a non-invasive, blood-test-based simple score that is able to reduce post-operative complications in patients with HCC.

Novel strategies to treat patients with ACLF have also been under development [24,25]. We are currently developing new strategies against HAV infections as acute insults [26,27]. In summary, the articles mentioned above offer a critical overview of ALF, ACLF and the related areas, and these medical conditions also play important roles in the COVID-19 era.

## Figures and Tables

**Figure 1:**
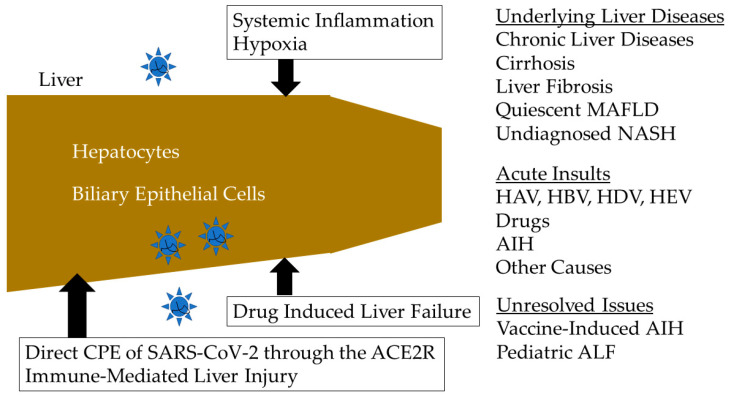
Acute liver failure (ALF) and acute-on-chronic liver failure (ACLF) in the coronavirus disease 2019 (COVID-19) era. CPE, cytopathic effect; SARS-CoV-2, severe acute respiratory syndrome coronavirus 2; ACE2R, angiotensin-converting enzyme 2 receptor; MAFLD, metabolic associated fatty liver diseases; NASH, non-alcoholic steatohepatitis; HAV, hepatitis A virus; HBV, hepatitis B virus; HDV, hepatitis D virus; HEV, hepatitis E virus; AIH, autoimmune hepatitis.

## Data Availability

The data might be available from the authors of the cited papers.
